# Biomechanical behavior of all-on-4 concept and alternative designs under different occlusal load configurations for completely edentulous mandible: a 3-D finite element analysis

**DOI:** 10.1007/s10266-024-00941-1

**Published:** 2024-04-30

**Authors:** Ayben Şentürk, Funda Akaltan

**Affiliations:** Department of Prosthodontics, Faculty of Dentistry, Ankara University, Ankara, Turkey

**Keywords:** Edentulous mandible, All-on-4, Angulated implants, Finite element analysis, Stress distribution

## Abstract

The aim of this study was to evaluate the effect of the All-on-4 design and 4 alternative implant-supported fixed prosthesis designs on stress distribution in implants, peri-implant bone, and prosthetic framework in the edentulous mandible under different loading conditions using three-dimensional finite element analysis (3D-FEA).Five different experimental finite element models (Model A (unsplinted 6), Model B (splinted 6), Model C (All-on-4), Model D (axial; 2 anterior, 2 posterior), and Model E (4 interforaminal)) were created. Three different loading conditions were applied (canine loading, unilateral I-loading, and unilateral II-loading). The highest minimum (Pmin) and the maximum (Pmax) principal stress values were acquired for cortical and trabecular bones; the highest von Mises (mvM) stress values were obtained for implants and metal frameworks. Model B and Model D showed the most favorable stress distribution. The All-on-4 design (Model C) also showed acceptable stress values close to those of Model B and Model D in the cortical and trabecular bones. In accordance with the stress values in the bone structure, the lowest stress values were measured in the implants and Co-Cr framework in Model B and Model D. The highest stress values in all structures were measured for unilateral loading- II, while the lowest values were found for canine loading. It was concluded that Model B and Model D experimental models showed better biomechanical performance in all structures. Furthermore, the use of a splinted framework, avoiding cantilevers, results in lower stress transmission. On the other hand, canine loading and unilateral loading-I exhibited the best loading conditions.

## Introduction

Tooth loss is a common problem in the elderly population [1]. For completely edentulous patients, full mouth reconstruction is essential to acceptable appearance, chewing function, and further good life qualities [2]. There are different treatment alternatives for completely edentulous patients. Edentulous patients can be rehabilitated with an overdenture prosthesis using 2–4 implants or with implant-supported fixed prosthesis using 6–8 implants. [3–7]. The traditional treatment procedure consists of a 3-piece fixed prosthetic design supported by at least 6 implants for a fully edentulous mandible [5, 8–10]. However, numerous studies over the past 10 years [5, 9, 11, 12] have shown that 2 well-angled and sufficiently long implants in the posterior region and 2 vertical anterior implants are suitable for the construction of full arch fixed restorations. The biomechanical analysis determined that a fifth or sixth implant was not essential for mechanical support and led to the development of the All-on-4 full-arch restoration strategy [5, 11]. It is possible to use 10–18 mm long implants with a diameter of 4/4.3 mm in the posterior and 3.75/4 mm in the anterior region [9, 13, 14]. Two anterior implants are positioned axially in the lateral or canine region [8, 15], and 2 posterior implants are positioned distally angled at 15–45 degrees in the second premolar or first molar area [1, 13, 16]. By placing distally angled implants in the interforaminal region, adequate primary stability is achieved, the anteroposterior spread of the implants is optimized, the length of the cantilevers is reduced, and oral rehabilitation can be performed with minimal surgical risk. [1, 2, 12, 14, 17–19]. Therefore, the All-on-4 design is an alternative to a fixed prosthesis supported by 6 implants in the mandible, a standard approach with good results [5, 8–10].

According to the original Branemark method, 4 or 6 implants should be placed between the mental foramina to support a bilateral distal cantilevered prosthesis [20]. Cantilever use causes biomechanical stress through prostheses and implants [21, 22]. Prosthetic failures such as peri-implant bone loss, screw loosening, or fracture are more common as the cantilever length increases [21–24]. Despite its drawbacks, cantilever construction with an acceptable size and shape can be considered an alternative treatment [24, 25]. It is recommended to reduce the cantilever as much as possible to decrease prosthetic complications [17]. In the All-on-4 technique, the cantilever length is shortened using distally angled implants, which allows a maximum cantilever length of up to two teeth [9, 26].

Misch [20] suggested that mandibular movements arising in the event of further separation from the mental foramina in fixed restorations adversely affected the prognosis of implants, and less bending forces occurred across the mandible by inserting implants between the mental foramina and splinting of the full-arch fixed restorations. The All on 4 technique is also a treatment protocol that generates less bending force in the mandible. Jensen et al. [27] placed 2 implants in the anterior region and 2 in the first molar region in a category defined as Class A under the All-on-4 classification for completely edentulous jaws when there were no anatomical restrictions in the posterior region. In this type of implant placement, fewer and more vertically placed implants reduce the cantilever length and complications related to implant angle. However, there is insufficient biomechanical research on this design, which is undesirable due to the effect of the bending force on the mandible.

In addition to the presence and length of cantilevers in full arch fixed restorations, another important issue is occlusion. Generally approved occlusion protocols have been successfully used for implant-supported prostheses [22, 28]. Implant-protected occlusion has been defined for implant-supported prostheses; this idea was developed to reduce the loads acting on the implants and to protect the implant against failure [20, 24]. It is obvious that occlusion is important for long-term success, especially in full arch fixed prostheses supported by a small number of implants.

The aim of this study was to evaluate the effect of the All-on-4 design and 4 alternative implant-supported fixed prosthesis designs on stress distribution in implants, peri-implant bone, and prosthetic framework in the edentulous mandible under different loading conditions using three-dimensional finite element analysis (3D-FEA). The hypothesis of this study is that different implant placements and different loading conditions will result in different stress distributions in the implants, peri-implant bone, and prosthetic framework.

## Materials and methods

**Fig. 1:**
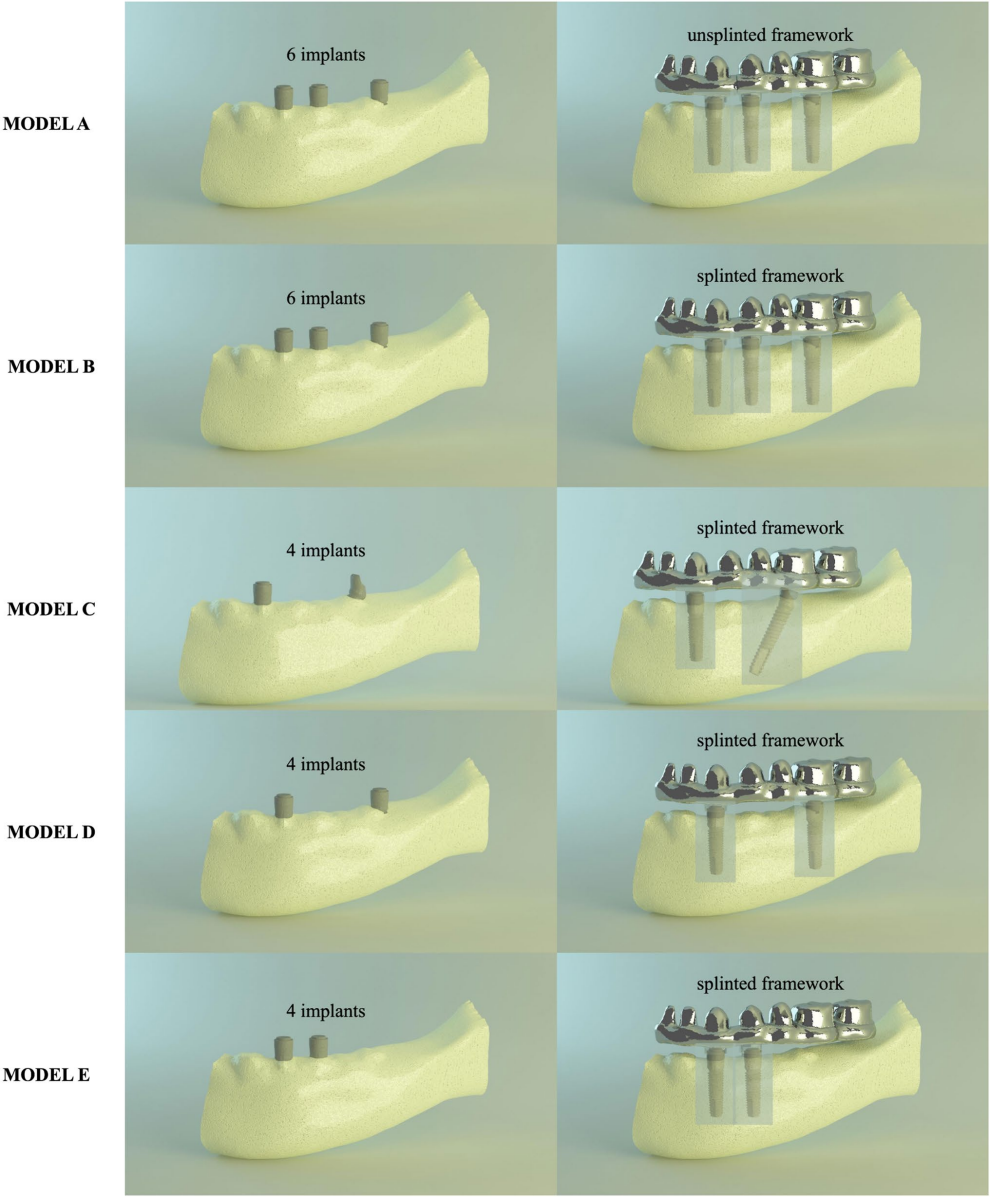
Five different experimental designs for the edentulous mandible

In this study, experimental models of the all-on-4 design and 4 alternative implant-supported fixed prosthetic designs (a total of 5 experimental finite element models) were created for a completely edentulous mandible. The different finite element models are shown in Fig. 1.

*Model A (unsplinted 6):* Three-piece unsplinted frameworks were planned on six implants. The implants were placed bilaterally in the canine, first premolar, and first molar regions. The unsplinted metal framework is designed in three pieces over implants in regions 34–37, 33–43, and 44–47.

*Model B (splinted 6):* A one-piece splinted framework was planned on six implants. The implants were placed bilaterally in the canine, first premolar, and first molar regions. A full-arch fixed prosthesis has been designed; All implants are splinted to each other with a metal framework.

*Model C (All-on-4):* A one-piece All-on-4 framework was planned on four implants. Mesial implants were placed axially in the canine region, and distal implants were placed distally angled to the 30-degree range of their apex in the first premolar region and their necks in the first molar region.

A full-arch fixed prosthesis has been designed; All implants are splinted to each other with a metal framework.

*Model D (axial; 2 anterior, 2 posterior):* A one-piece framework was planned on four implants. All implants were positioned axially and bilaterally in the canine and the first molar regions. A full-arch fixed prosthesis has been designed; All implants are splinted to each other with a metal framework.

*Model E (4 interforaminal):* One-piece framework was planned on four implants. The implants were placed in the canine and the first premolar regions. A full-arch fixed prosthesis has been designed. All implants are splinted to each other with a metal framework.

The edentulous mandible was modeled using Rhinoceros and VRMESH software programs, with a 2 mm cortical bone layer covering the trabecular bone. MRI or CT images were not used to create the models. Thus, standardization was ensured between the FEA models and focused only on the stresses caused by different treatment plans. Implants and prosthetic components were scanned using an optical scanner (Activity 880, Smart Optics Sensortechnick), and the data was reconstructed using VRMESH software. All structures were modeled using the Rhinoceros 4.0 program. A standard cylindrical hexagonal implant design was used in all treatment planning. Axial implants were modeled as having a 4 mm diameter and 12 mm length, while angled implants were modeled as having a 4 mm diameter, 18 mm length, and a 30º angle. The cortical bone thickness is 2 mm in the neck area of the implants. The abutments and implants were managed as a whole; the abutments were 4 mm in diameter and 4 mm in length; and the abutments were angled 0-degree in axial implants; the abutments with a 30-degree angle related to the long axis of the implant were used in distally angled implants. The porcelain superstructure was modeled on the Co-Cr framework in the prepared tooth form. The frame height and the porcelain superstructure were estimated to be 8 mm and 2 mm, respectively. For all groups except the fifth, the length of the cantilever was designed to be 10 mm. For the fifth group, it was measured at 19 mm from the first molar and 33 mm from the second molar. The implant-bone interface was considered to be fully osseointegrated.

The number of elements and nodes in the final models is shown in Table 1. The models are fixed at the bottom and sides of the bone so that they have zero movements in the DOF (Degree of Freedom). All models were accepted as linearly homogenous, elastic, and isotropic. Elastic modulus and Poisson’s ratios of the materials were obtained from the most commonly used values of researchers in the previous studies [6, 8, 29–31] and are presented in Table 2.

**Table 1:** The number of elements and nodes for all models

FEA Models	Elements	Nodes
Model A (unsplinted 6)	1,224,836	230,643
Model B (splinted 6)	1,233,431	231,476
Model C (All-on-4)	1,070,418	203,909
Model D (axial; 2 anterior, 2 posterior)	1,067,103	202,389
Model E (4 interforaminal)	1,068,665	202,644

*FEA* finite element analysis

**Table 2:** Mechanical properties of the materials

Material	Elasticity modulus (MPa)	Poisson ‘s ratio	Reference
Cortical bone	13,700	0,30	[8, 29, 31]
Trabecular bone	1,370	0,30	[8, 29, 31]
Titanium	110,000	0,30	[30]
Co-Cr	218,000	0,30	[6]
Ceramics	70,000	0,22	[6]

**Fig. 2:**
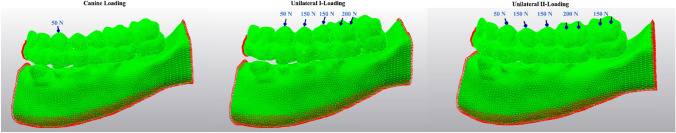
Three different loading conditions

The finite element models were exported to ALGOR FEMPRO software (Algor) for 3D static analysis. In the present study, three different loading conditions were applied (Fig. 2):

- *(1) canine-loading;* 50 N force was applied unilaterally to the canine at an angle of 45º,
- *(2) unilateral I-loading*; 50 N, 150 N, 150 N, and 200 N forces were applied unilaterally to the canine, first premolar, second premolar, and first molar, respectively, at an angle of 45º,
- *(3) unilateral II-loading*; 50 N, 150 N, 150 N, 200 N, and 150 N forces were applied unilaterally at an angle of 45 degrees to the canine, first premolar, second premolar, first molar, and second molar, respectively. To simulate the relieved occlusal contacts in the cantilevered second molar region, a force of 150 N, less than the 200 N applied to the first molar region, was applied. The ideal clinical conditions were intended to be simulated without disregarding the relieved occlusal contacts in the cantilever region.

The analysis results were produced numerically and transformed into visual results with color codes. The highest minimum (Pmin) and the maximum (Pmax) principal stress values were obtained for cortical and trabecular bones; the highest von Mises (mvM) stress values were achieved for implants and metal frameworks.

## Results

### Stresses in cortical bone

**Fig. 3:**
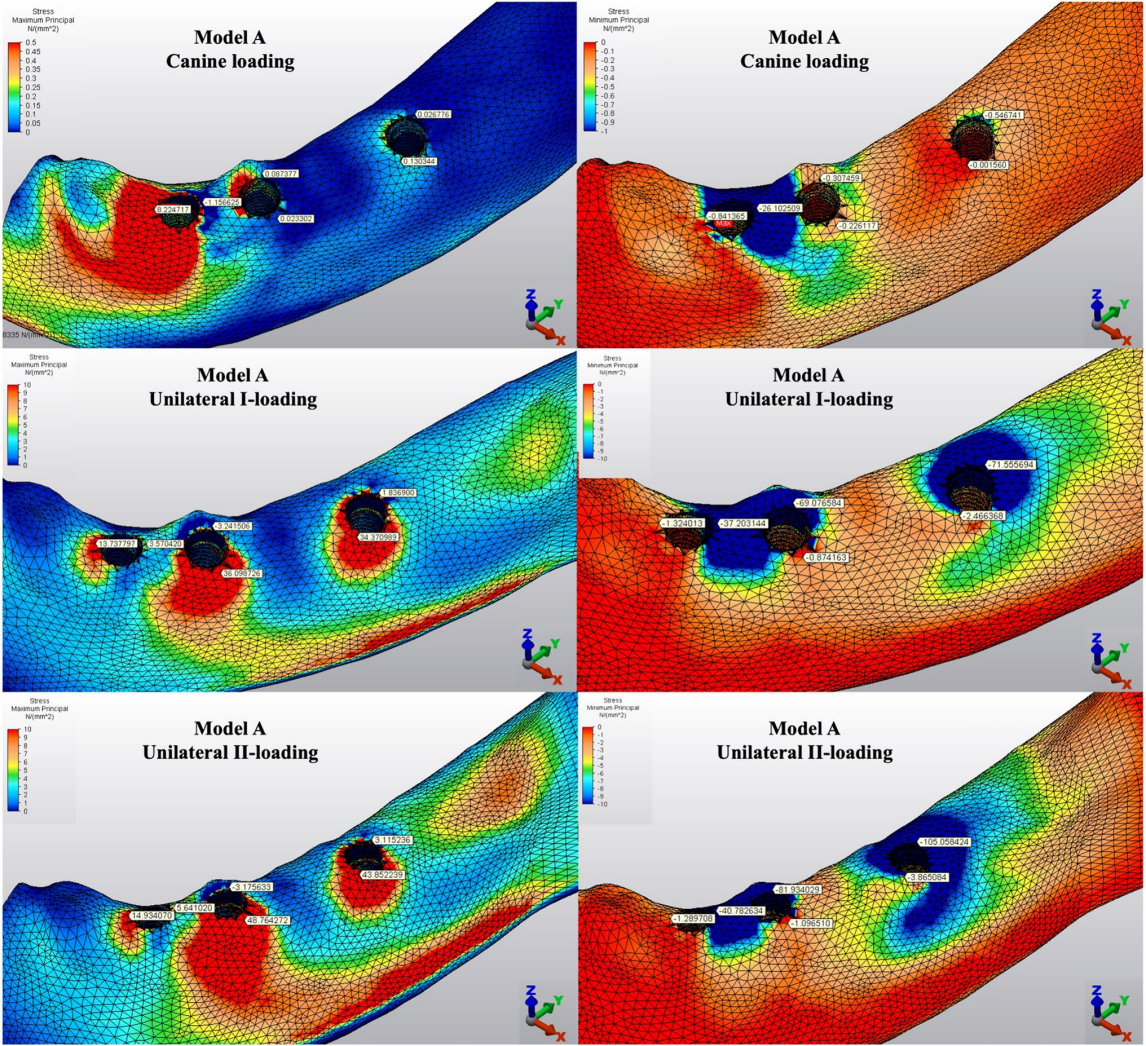
Distribution of Pmin and Pmax values of Model A on cortical bone

**Fig. 4:**
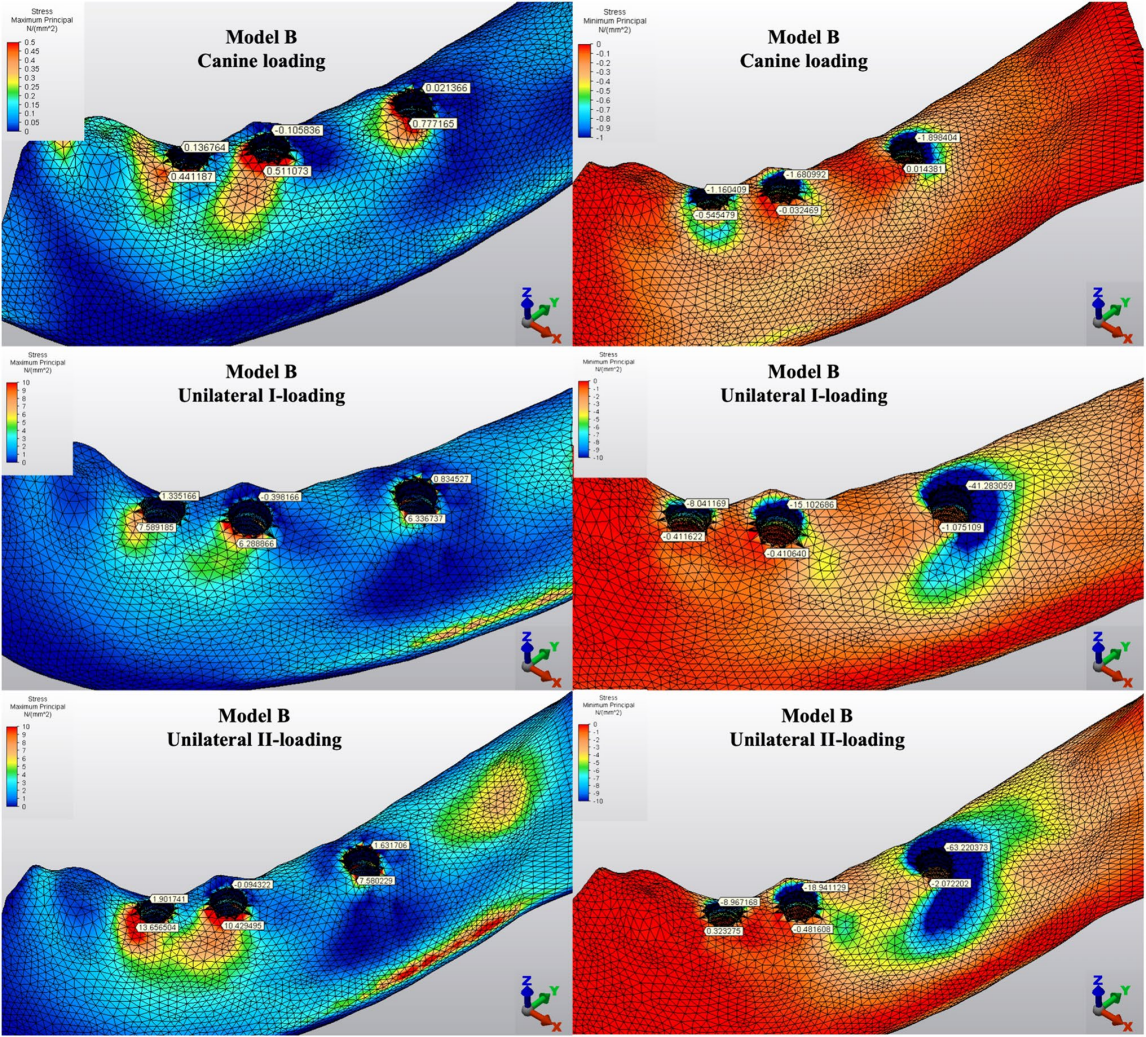
Distribution of Pmin and Pmax values of Model B on cortical bone

**Fig. 5:**
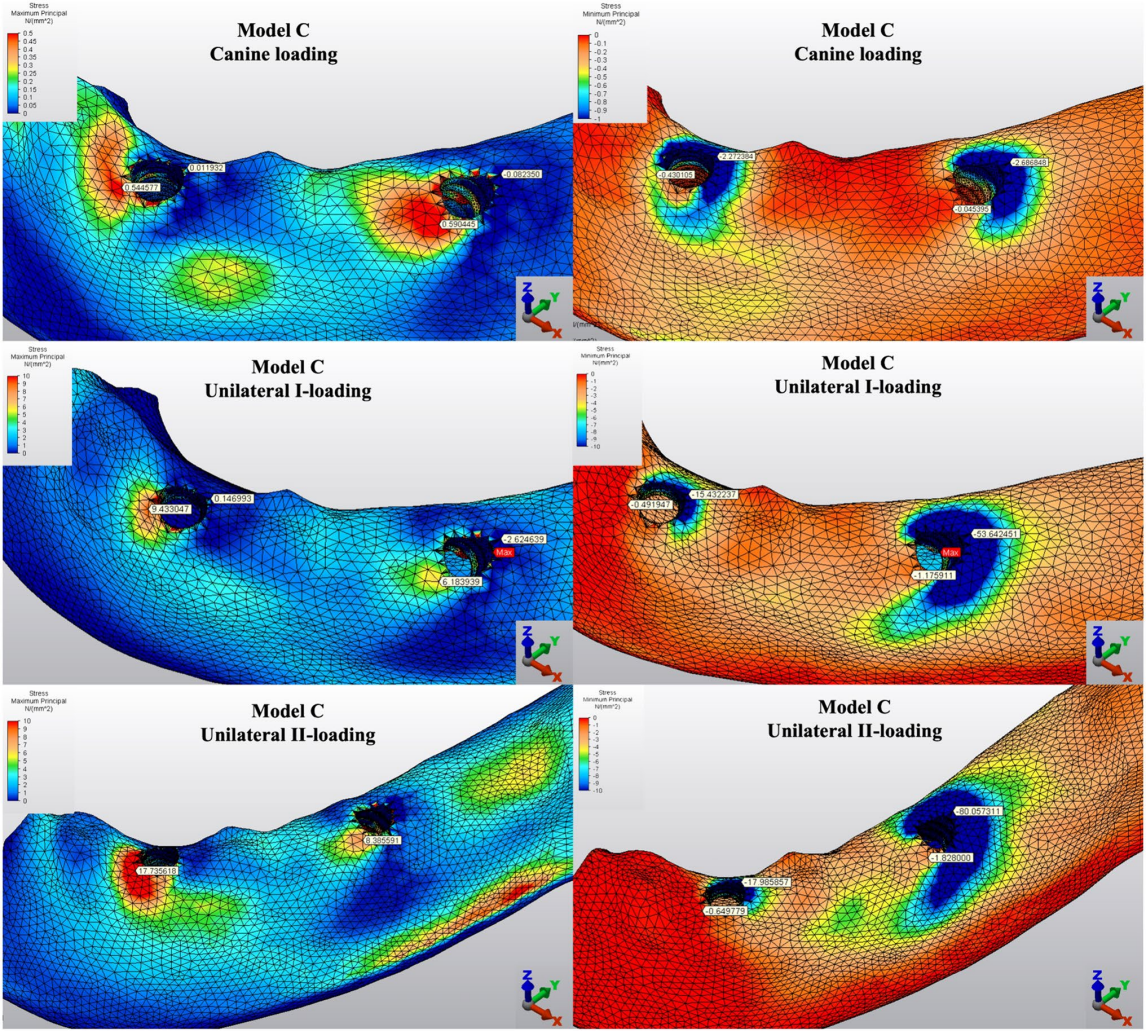
Distribution of Pmin and Pmax values of Model C on cortical bone

**Fig. 6:**
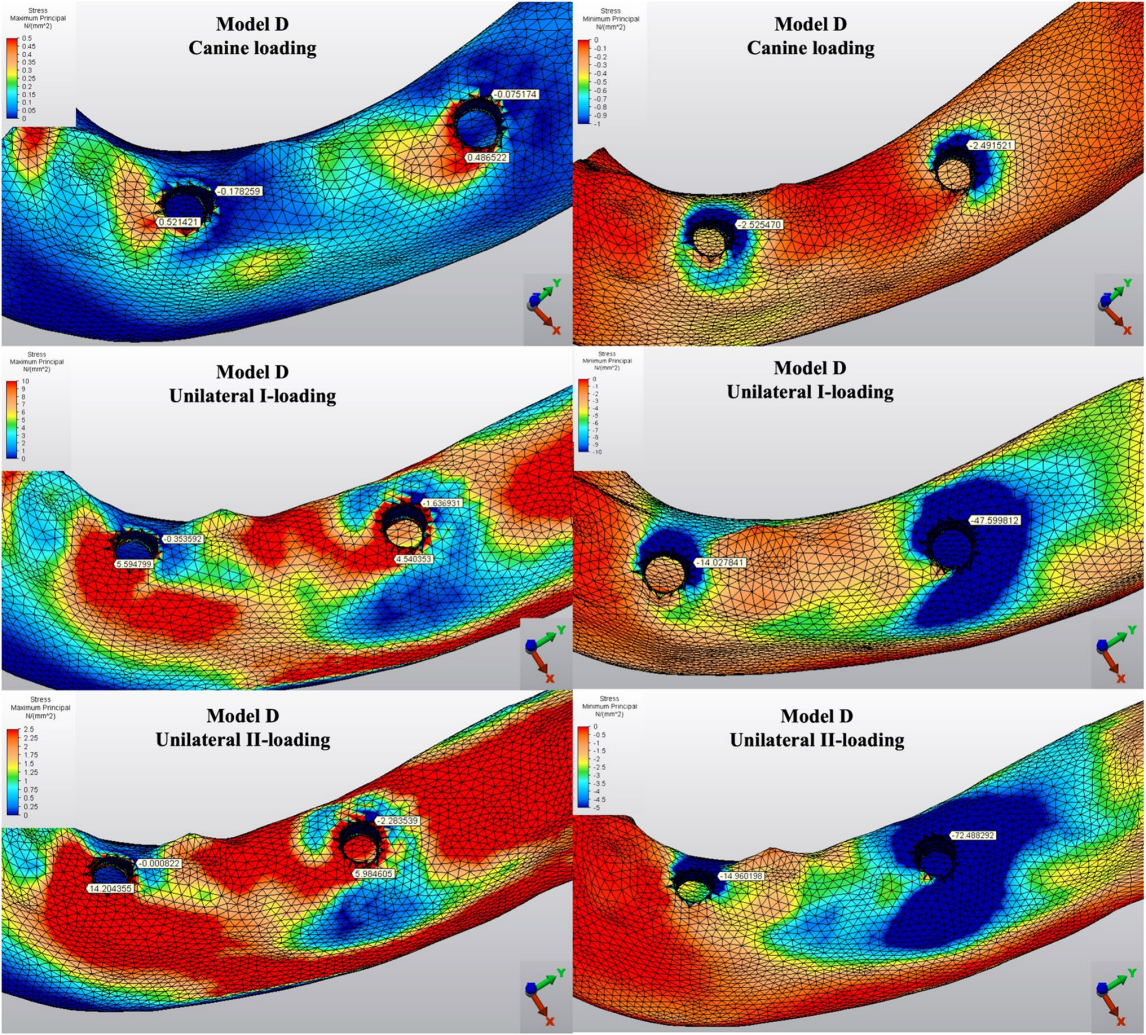
Distribution of Pmin and Pmax values of Model D on cortical bone

**Fig. 7:**
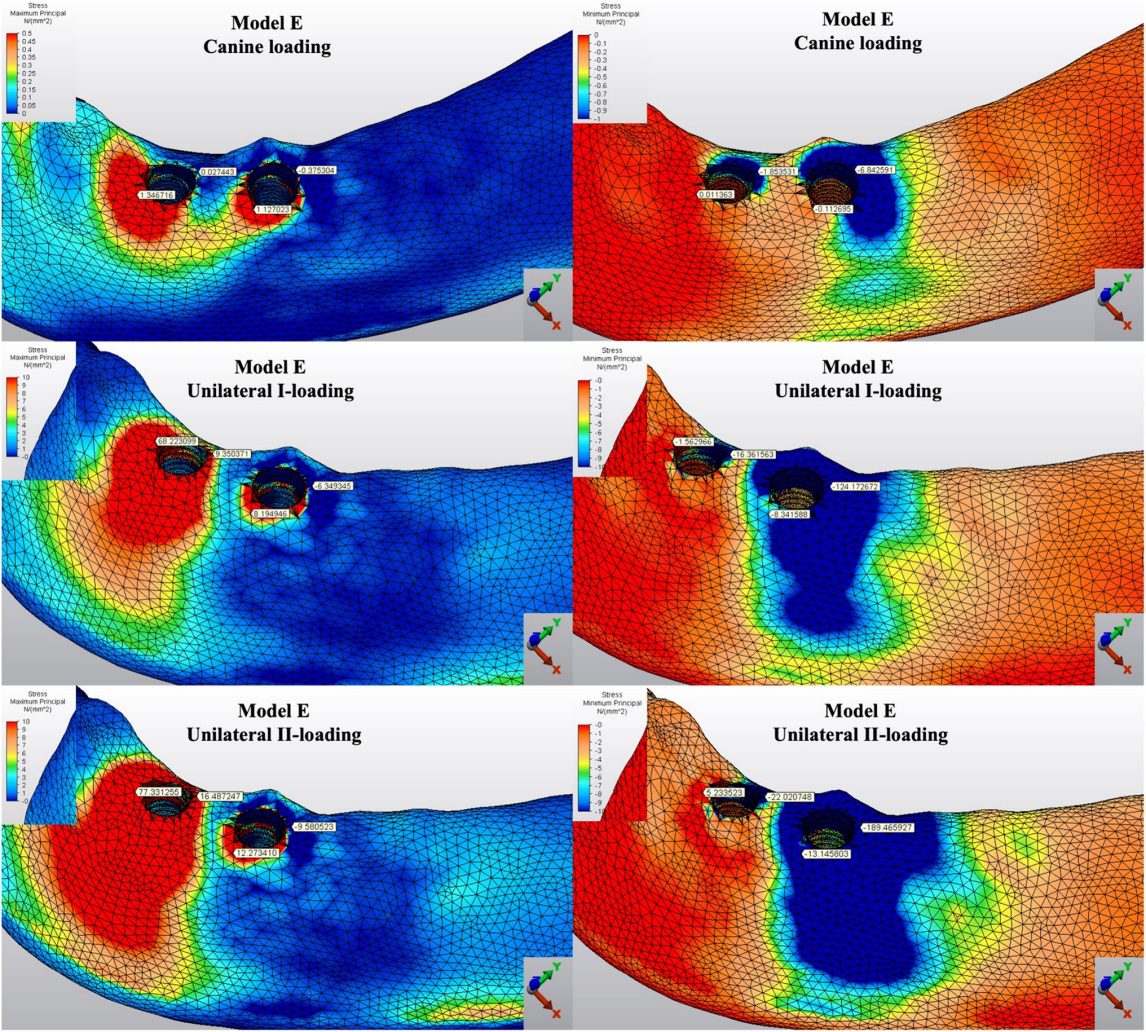
Distribution of Pmin and Pmax values of Model E on cortical bone

The Pmin and Pmax values in cortical bone are shown in Figs. 3, 4, 5, 6, 7 for each experimental design (Model A (Fig. 3), Model B (Fig. 4), Model C (Fig. 5), Model D (Fig. 6), and Model E (Fig. 7)).

#### Minimum principal stresses

The highest Pmin values in the bone were concentrated around the implant neck in all finite models. For the canine-loading condition, the highest Pmin cortical bone value (− 26.10 MPa) was observed around the anterior implant in Model A (unsplinted 6) (Fig. 3). Splinting of 6 implants (Model B) (Fig. 4) considerably reduced the Pmin values, particularly for the canine and premolar regions, under all loading conditions. The highest Pmin values (− 63.22 to 189.47 MPa) for cortical bone were measured under unilateral I and II loading conditions around the most distal implants. Model A (unsplinted 6) showed a higher Pmin value (− 105.06 MPa) than Model E (4 interforaminal) (− 189.47 MPa), while other groups showed similar stress values. All Pmin values were below the strength capacity of the bone (yield strength: 170 to 190 MPa). However, the Pmin value for distal implants under the unilateral II loading condition was -189.47 MPa, quite similar to the final strength of the cortical bone for Model E (4 interforaminal) (Fig. 6).

#### Maximum principal stresses

The highest Pmax values in the cortical bone were achieved around the neck of the implant and were observed under unilateral II and I loading conditions, respectively. For the canine-loading condition, the highest Pmax value (8.22 MPa) for the cortical bone was observed around the canine implant in Model A (unsplinted 6) (Fig. 3). Splinting 6 implants (Model B) greatly reduced the Pmax values under all loading conditions. In Model E (4 interforaminal) (Fig. 7), the highest Pmax value (77.33 MPa) was observed around the anterior implant region under unilateral loading conditions. However, the maximum principal stress values were below the strength capacity of the cortical bone (yield strength: 100 to 130 MPa) for all groups.

### Stresses in the trabecular bone

**Fig. 8:**
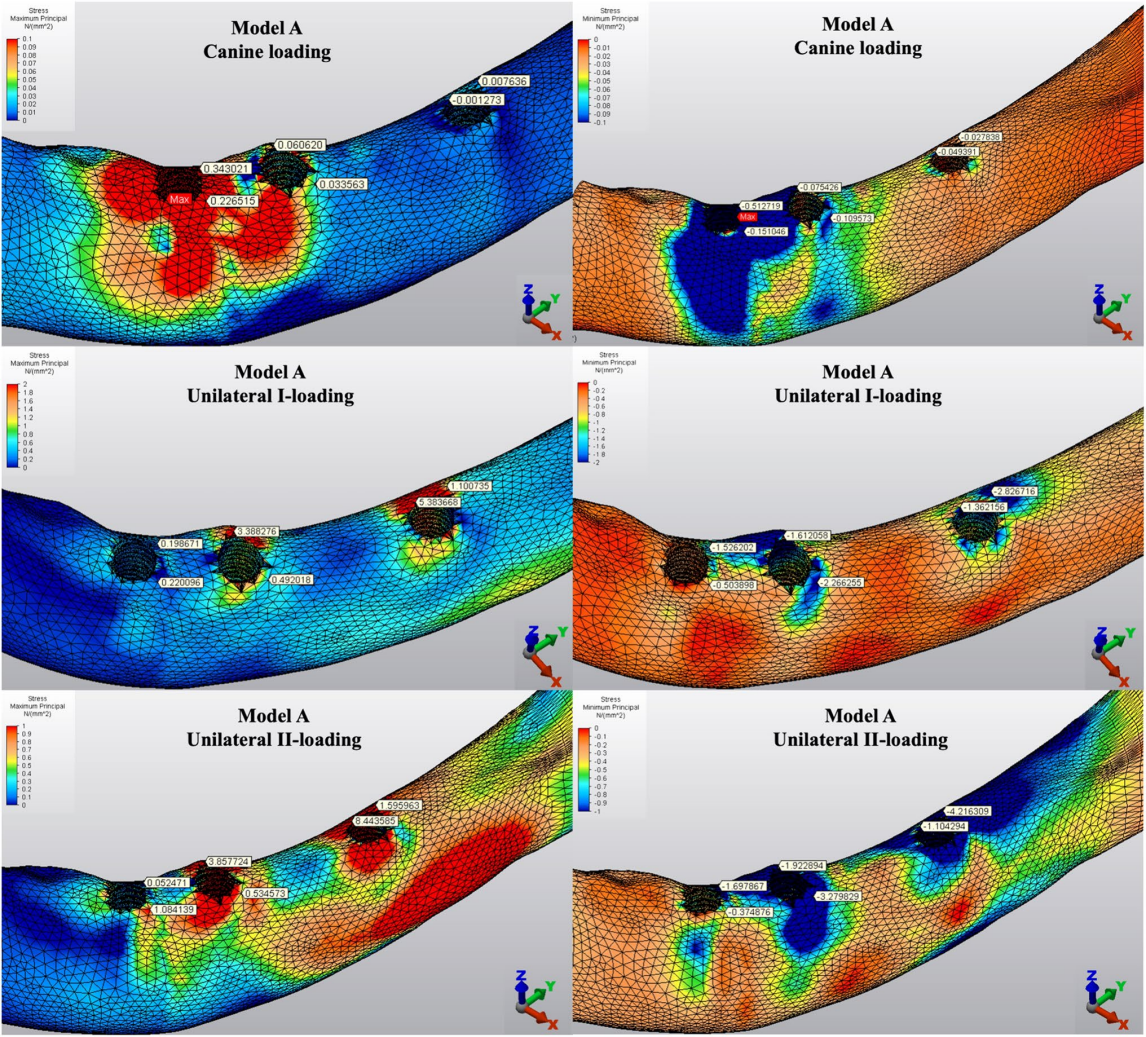
Distribution of Pmin and Pmax values of Model A on trabecular bone

**Fig. 9:**
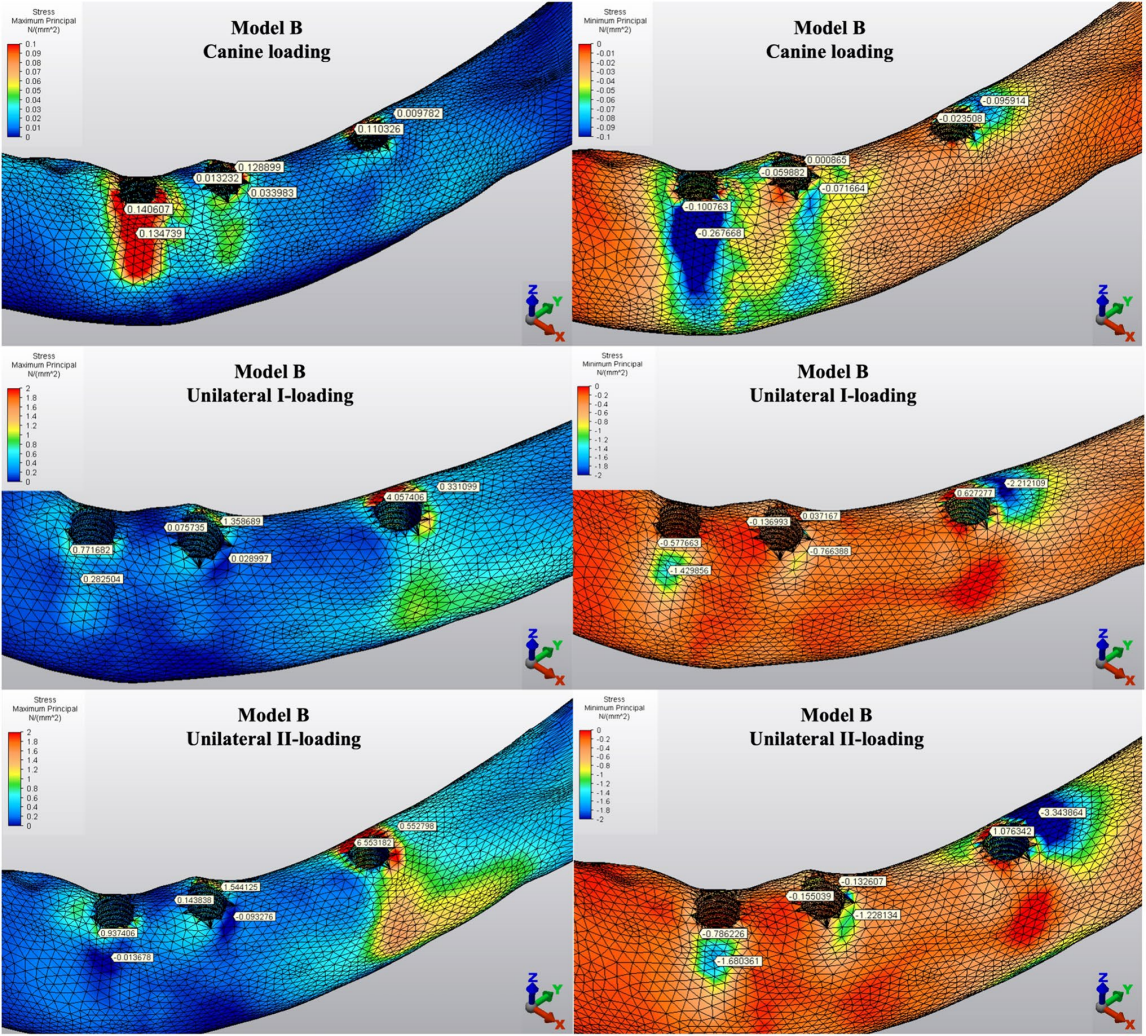
Distribution of Pmin and Pmax values of Model B on trabecular bone

**Fig. 10:**
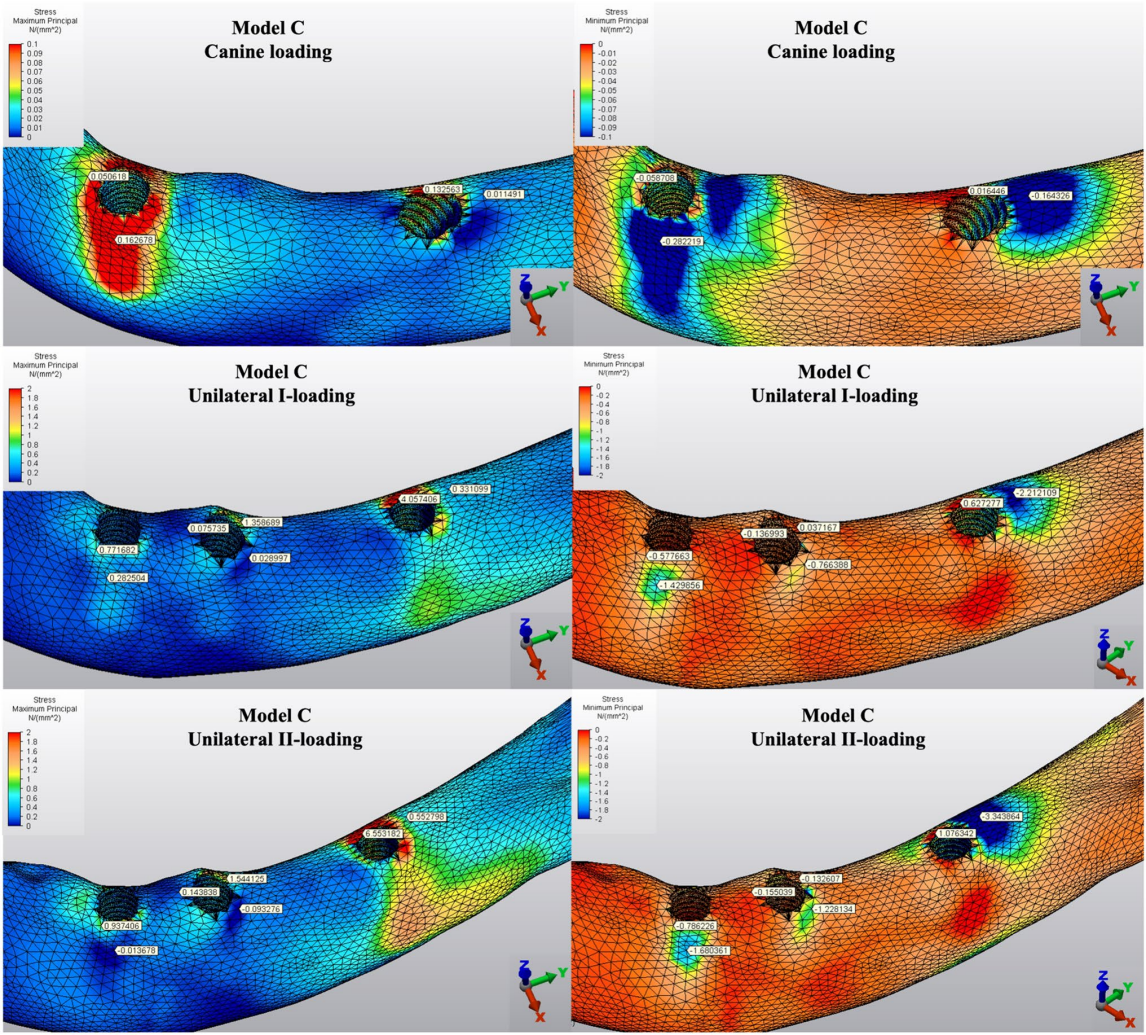
Distribution of Pmin and Pmax values of Model C on trabecular bone

**Fig. 11:**
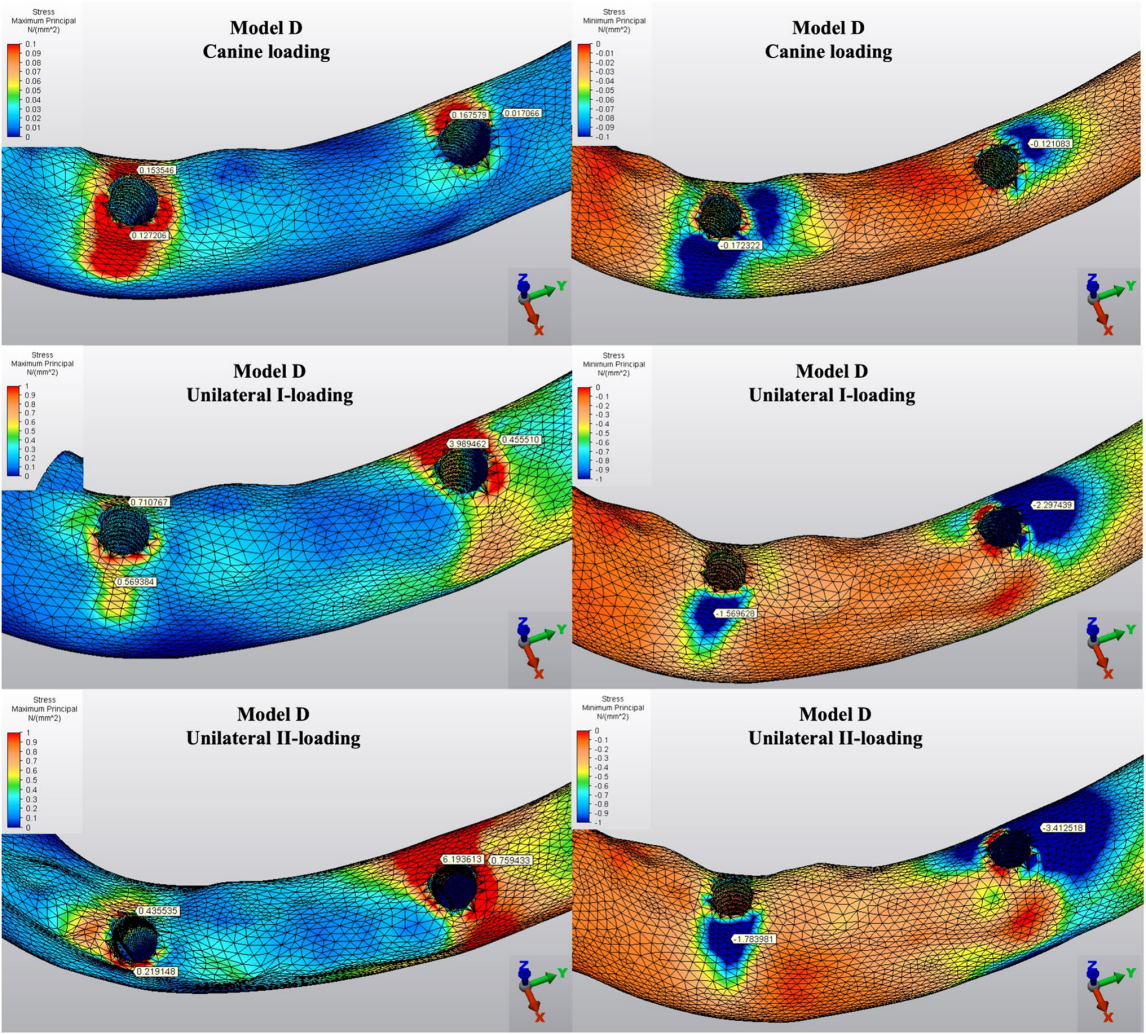
Distribution of Pmin and Pmax values of Model D on trabecular bone

**Fig. 12:**
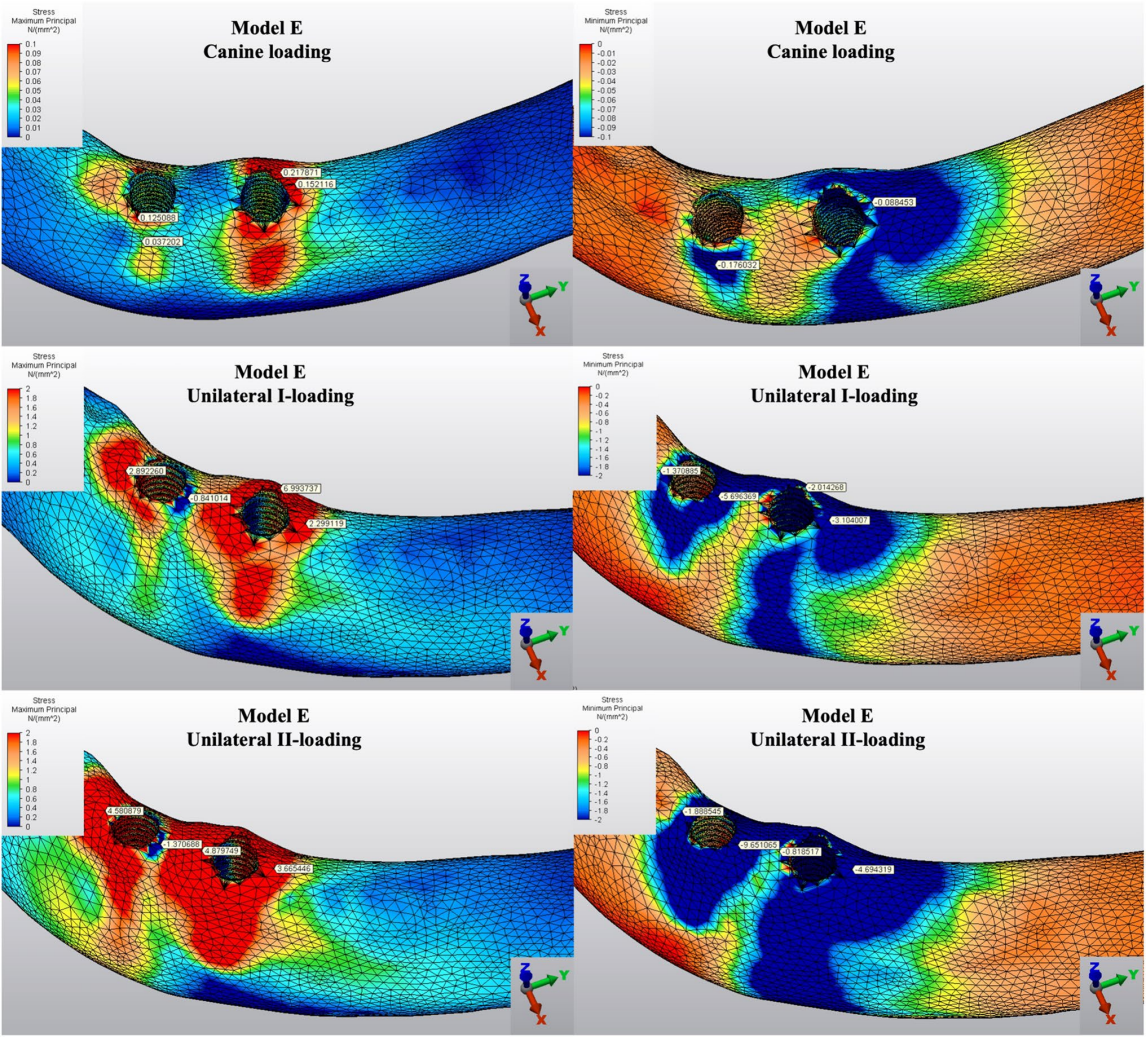
Distribution of Pmin and Pmax values of Model E on trabecular bone

The Pmin and Pmax values in trabecular bone are shown in Figs. 8, 9, 10, 11, 12 for each experimental design (Model A (Fig. 8), Model B (Fig. 9), Model C (Fig. 10), Model D (Fig. 11), and Model E (Fig. 12).

#### Minimum principal stresses

The highest Pmin values observed in the trabecular bone were concentrated around the neck of the implant. All models exhibited the lowest Pmin values under canine loading. The highest stress values (− 0.05 to − 0.51 MPa) in the trabecular bone were found around the anterior implant for all models under canine-loading. Under unilateral loading conditions, the highest Pmin stresses were concentrated around the anterior implant only for Model E (4 interforaminal) (Fig. 12). For the other models, the highest Pmin values in the trabecular bone occurred around the most distal implants. Model B (splinted 6) (Fig. 9) and Model D (axial; 2 anterior, 2 posterior) (Fig. 11) showed the lowest Pmin values under all loading conditions. In the other groups, Pmin values were close to or above − 5 Mpa, which is the strength capacity of the bone, particularly for unilateral II loading conditions.

#### Maximum principal stresses

The highest Pmax values observed in the trabecular bone were concentrated around the neck of the implant. In canine-loading, the highest Pmax value (0.34 MPa) was localized mainly around canine implants for Model A (unsplinted 6) (Model 8), while stress levels were distributed over all implants for the other models. The highest Pmax values were observed under unilateral loading conditions around the distal implants. In all models, the unilateral II loading condition showed the highest Pmax stress values (4.88 to 8.44 MPa), and these values were above the bone strength capacity or very close to the maximum strength potential (yield strength: 5 MPa). Under unilateral loading I, stress values were above the bone strength capacity for Model A (unsplinted 6) (5.38 MPa) (Fig. 8) and Model E (4 interforaminal) (Fig. 12) (6.99 MPa).

### Stresses for implants

**Fig. 13:**
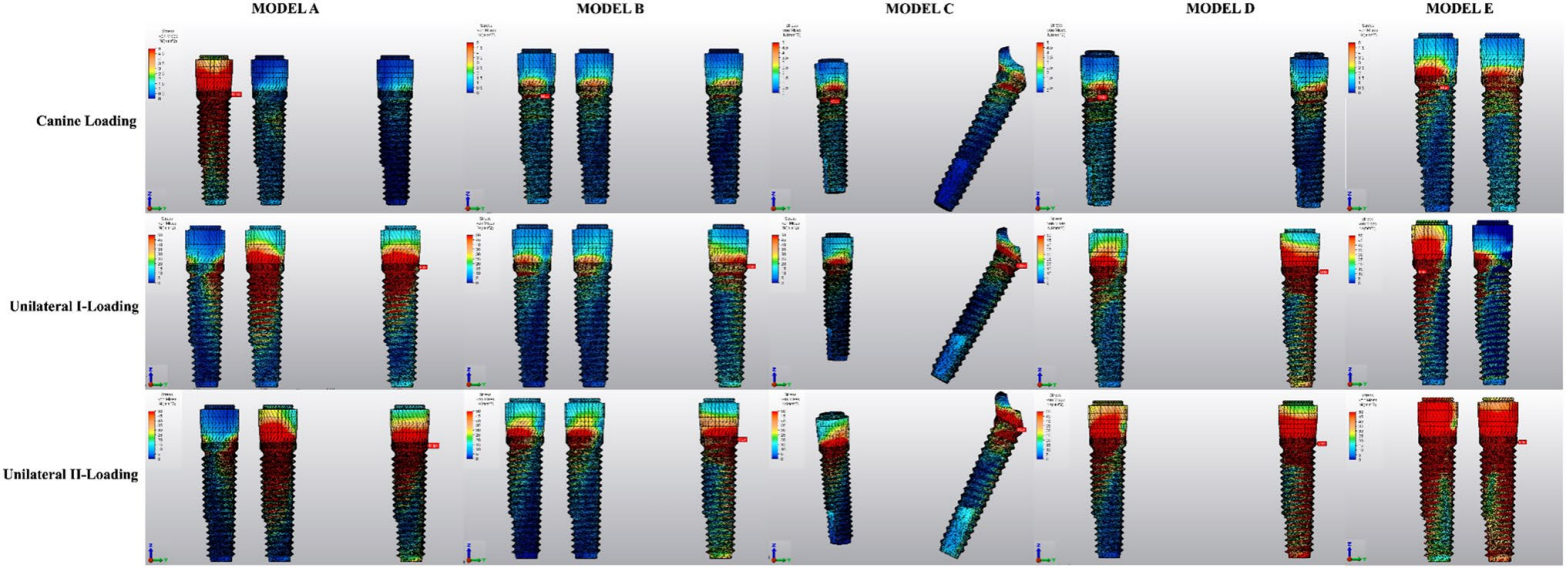
Distribution of von Mises stresses in implants

The highest von Mises stress values for implants were found in the grooves of the neck region. (Fig. 13) In all groups, the lowest von Mises stress values of implants (17.10 to 81.50 MPa) were observed under canine-loading, and the highest values (273.61 to 699.56 MPa) were obtained under unilateral II loading. For the canine-loading condition, the highest von Mises stress values were observed in canine implants for all groups. Under unilateral loading conditions, the highest Von Mises stress values (171.98 to 699.56 MPa) were concentrated in the most distal implants, with values that were very close to the strength of the implants.

Model A (unsplinted 6) and Model E (4 interforaminal) caused the highest von Mises stress values in implants under all loading conditions, and the most appropriate stress distribution was observed for Model D (axial; 2 anterior, 2 posterior) (8.71 to 273.61 MPa) (Fig. 13).

### Stresses for metal frameworks

The maximum von Mises stress values for the framework material were measured from the abutment-framework connections. The lowest von Mises stress values (0.82 to 2.98 MPa) were attained under the canine-loading condition, while the highest values were observed for the unilateral II loading condition (55.57 to 335.08 MPa). Splinting 6 implants significantly lowered the maximum stress values of von Mises.

**Fig. 14:**
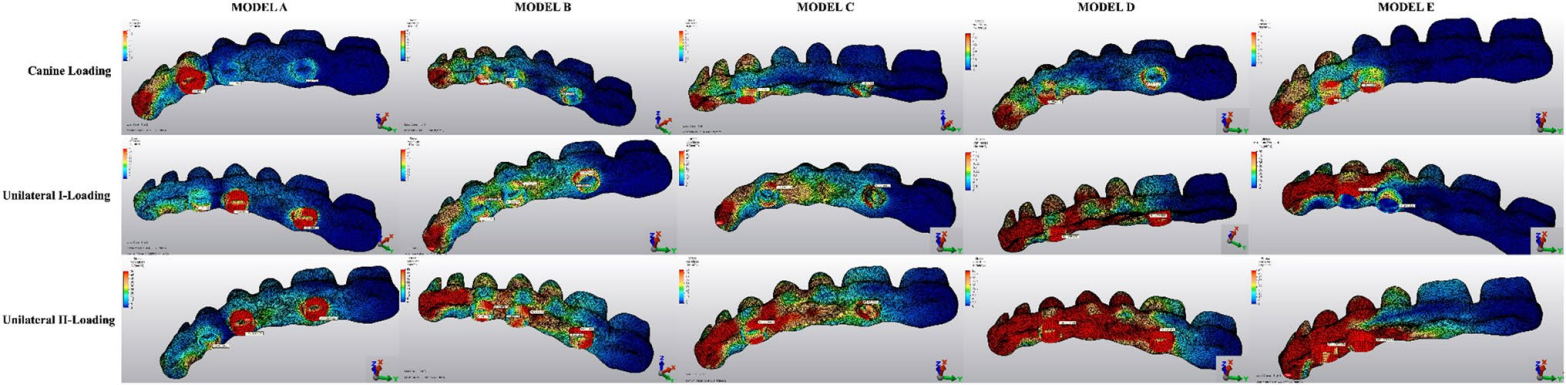
Distribution of von Mises stresses in metal framework

The highest von Mises stress values through the frameworks were observed for Model E (4 interforaminal) (335.08 MPa), Model C (All-on-4) (188.76 MPa), and Model A (unsplinted 6) (172.60 MPa), respectively, under unilateral loading conditions. Model B (splinted 6) (90.58 MPa) and Model D (axial; 2 anterior, 2 posterior) (108.35 MPa) rendered the lowest von Mises stress values for the metal framework (Fig. 14).

## Discussion

The investigation of successful treatment plans with fewer implants continues.. Methods that are biomechanically beneficial and more efficient and do not require surgical procedures are studied in-vivo and in-vitro, and new treatment proposals are put on the agenda. Results of long-term clinical trials have shown that fixed prostheses on four implants have comparable success levels to prostheses on multiple implants.

The study of stress transmitted to implants, bones, and structures by All-on-4 and alternative experimental models under various loading conditions in which occlusion differences can be assessed, the interpretation of how stress differs between experimental models, and the evaluation of discrepancies caused by the presence or absence of cantilever and splinting were the objectives of this research. The hypothesis of the study was accepted. Different implant placements and different loading conditions caused different stress distributions in the implants, peri-implant bone, and prosthetic framework.

In this study, the highest Pmin stress value of 189.47 MPa and Pmax stress value of 77.33 MPa in cortical bone among all experimental models was measured in Model E (4 interforaminal), supported by a total of 4 implants in the canine and first premolar regions. Model B (splinted 6), Model C (all-on-4), and Model D (axial; 2 anterior, 2 posterior) experimental models exhibited similar stress values in the cortical bone, but the lowest stress values were observed in Model B, in which the axial 6 implants were splinted with a metal framework. Previous studies have reported that prostheses supported with 6 implants show a more favorable stress distribution than prostheses supported with 4 implants [29, 32]. The stress values in the trabecular bone, metal framework, and implants were consistent with the cortical bone values in all experimental models; the most favorable stress distribution was observed in Model B in all structures examined.

The All-on-4 procedure, a common approach and an alternative to fixed prostheses on 6 implants in the mandible, has shown favorable results [5, 8, 9, 33]. In this study, the all-on-4 experimental model (Model C) exhibited a stress distribution close to that of Model B, in which the metal framework supported by 6 implants was splinted. However, the Model D experimental model supported with 4 axial implants exhibited a more favorable stress distribution than the All-on-4 model (Model C). In Model C, the distal implant placed at a 30-degree angle showed higher stress values than the distal axial implant in Model D. Although there are different results for the stress values of angled distal implants compared to axial implants [1, 10, 15, 16, 34–36] in general, in the All-on-4 concept, the angled distal implant reduces the stresses that will occur due to the reduction of the cantilever length in the prosthetic framework and causes appropriate stress distribution [10]. Decreasing cantilever length reduces the stress values transferred to the bone [32, 33]. Ozan and Kurtulmuş [37] investigated the effect of implant angulation and cantilever length on stress distribution and concluded that decreasing cantilever length due to implant angulation reduces stress on bone and prosthetic components.

In this study, the cantilever length was 10 mm for all groups except Model E (4 interforaminal) and was consistent with the ideal cantilever length recommended in other studies [37–39]. Since the cantilever length in Model E (4 interforaminal) is above safe limits (Unilateral II-loading condition), the stress values observed in the implants and prosthetic substructure in the bone are quite high and above the bone strength limit. It is not surprising that such high stress values are observed, especially under unilateral II loading conditions. On the other hand, although the cantilever length was within the ideal limits (10 mm) in Model A (unsplinted 6), the observed stress values were quite high. Therefore, it was assumed that splinting the implants when using cantilevers would make the stress distribution more favorable.

In the rehabilitation of the fully edentulous mandible with fixed implant retained prostheses, a minimum of 4 implants are required in the interforaminal region [40]. Akça and Küçükkurt (2023) conducted a study on stress distribution in implant-supported fixed prostheses with different numbers of implants, stating that a configuration of interformainal 4 axial implants was the most advantageous design in many aspects [41]. Similarly, Elawady et al. (2023) emphasized that interformainal 4 axial implants in screw-retained full arch prosthesis does not influence the implant survival nor the peri-implant marginal bone loss [42]. In light of these informations, in this study, a fixed implant-supported prosthesis model (Model E) with 4 implants placed in the interforaminal region was created. The stress values in the implants were measured under three different loading conditions. Under the Unilateral I-loading condition, the effect of a cantilever extension of 2 teeth wide was measured; under the Unilateral II-loading condition, the effect of a cantilever extension of 3 teeth wide on the implants was measured. Although the acceptability of a cantilever extension of 3 teeth wide under clinical conditions may not be very high, assessment was conducted in all possible scenarios. Both 2-teeth-wide and 3-teeth-wide cantilever extensions exhibited considerably high stress values for Model E.

Misch [20] reported that in fixed restorations, mandibular movements that occur when the implants are placed more distal than the foramina negatively affect the prognosis of the implants; in full arch fixed restorations, less bending strength occurs in the mandible when the implants are placed between the mental foramina. Likewise, in a study conducted by Charkawi et al. [43], they evaluated mandibular flexure in patients who had been using long-span rigid mandibular fixed prostheses for 10–15 years. They found a deviation in mandibular flexure values between splinted and non-splinted prostheses. They suggested that splitting the full arch prosthesis could prevent the negative consequences of mandibular flexure on restorations [43]. Caggiano et al. also emphasized that the biomechanical effects of mandibular flexion on fixed restorations are debated; they suggested that prospective clinical and radiological observational studies should be conducted to evaluate the potential short-, medium-, and long-term consequences of MF [44]. In this study, cortical bone stress values for Model B (splinted 6) and Model D (axial; 2 anterior, 2 posterior), in which distal implants were placed in the molar region and the structures were splinted, were found to be quite appropriate. Splinting is one of the most important factors in stress distribution. However, due to the limitations of finite element analysis in this study, the mandibular flexure effect could not be evaluated. The results obtained in Model B and Model D may be the opposite in the clinical situation due to the muscle activity and mandibular flexure. This aspect should not be overlooked in the evaluation of the results. The lack of evaluation of the effect of mandibular flexure on the results is among the limitations of this study.

Previous studies investigating the biomechanical behavior of implant-supported fixed full arch restorations have used different loading conditions [8, 10, 31]. In this study, the masticatory forces on natural teeth [45, 46] were used to reflect the masticatory forces on fully dentated individuals. The maxilla was considered fully dentate. Three specific loading scenarios were designed to simulate a canine-protected and unilaterally balanced occlusion that is accurate for dental individuals and also to evaluate the effect of the cantilever in the second molar region. In *canine loading*, a force of 50 N was applied obliquely at the incisal end of the canine teeth. In the *unilateral loading I* scenario, 50 N, 150 N, 150 N, and 200 N forces were applied to the canine, first premolars, second premolars, and first molars. In the *unilateral loading II* scenario, a force of 150 N was applied to the second molar instead of 200 N. The reason for applying a lower force to the second molar region compared to the first molar region is the deliberate reduction of occlusal contacts in the cantilever region.

Canine loading, unilateral loading I, and unilateral loading II conditions exhibited increasing stress values in all experimental model groups, implants, peri-implant bone, and metal substructure, respectively. In this context, the results of this study support the view that limiting the working side contacts in the anterior region in the arrangement of occlusal relationships in implant-supported fixed prostheses will lead to a reduction in stress. Especially canine loading and unilateral I loading conditions showed favorable stress distribution. Similarly, Bozyel and Taşar [47] concluded that canine-guided occlusion is a more suitable occlusion type for patients undergoing implant treatment.

This study simulated clinical conditions as accurately as possible; however, 3D-FEA studies have some limitations regarding bone type (cortical bone was considered 2 mm), boundary conditions, not evaluating mandibular flexure, level of osseointegration (100% osseointegrated), and material properties (homogenic and isotropic). These limitations were the same for all 5 experimental models in this study, as the aim of this study was not to report accurate stress values but to compare the biomechanical behavior of different implant configurations. This 3D-FEA study demonstrated the stress distribution of implants placed at different sites, implant angulation, the presence of a cantilever, splinting, and different loading conditions, which should be supported by long-term clinical studies.

## Conclusion

Based on the methods used in this study and the subsequent analysis of the results, it was possible to conclude that:

1. The configuration of the splinted 6 implants (Model B) and the axially placed configuration of the 2 anterior and 2 posterior implants (Model D) showed a better distribution of stress for the fully edentulous mandible compared to other experimental models.
2. The All-on-4 design (Model C) demonstrated appropriate biomechanical behavior. The use of angled (30-degree) implants did not affect stress values as much as the length of the cantilever, the splinting, and the configuration of the occlusal load.
3. The splinted framework design (Model B) resulted in a substantial reduction in stress values relative to the non-splinted framework design (Model A).
4. Increased cantilever length (Model E; 4 interforaminal) caused the most harmful stress levels in peri-implant bone, implants, and metal frameworks, particularly under unilateral loading.
5. Canine protected occlusion is a convenient occlusion protocol for fixed full-arch implant-supported restorations, but higher stress values around the canine implant can be achieved for unsplinted frameworks.
6. The elimination of occlusal loads on the second molar or the termination of the dental arc in the first molar can reduce stress levels around the posterior implants when planning unilaterally balanced occlusion.

These results underscore the importance of implant configuration, framework design, and occlusal load management in optimizing stress distribution and ensuring the long-term success of implant-supported restorations in fully edentulous mandibles. Therefore, Model B, with a single-piece splinted framework demonstrated the best results for canine-loading.

## Clinical relevance

Angled implant placement did not affect stress generation as much as the presence of a cantilever and splinting of the framework. Therefore, it is very important to splint the framework and reduce the cantilever length with implant placement in the posterior region or angled implant placement to make the cantilever as short as possible in the treatment of the edentulous lower jaw with a full arch fixed implant supported prosthesis.

## Data Availability

Data sets analyzed during the current study are available from the corresponding author on reasonable request.
